# Interaction of polyamines, abscisic acid and proline under osmotic stress in the leaves of wheat plants

**DOI:** 10.1038/s41598-018-31297-6

**Published:** 2018-08-27

**Authors:** Magda Pál, Judit Tajti, Gabriella Szalai, Violeta Peeva, Balázs Végh, Tibor Janda

**Affiliations:** 1 Department of Plant Physiology, Agricultural Institute, Centre for Agricultural Research, Hungarian Academy of Sciences, 2462 Martonvásár, POB 19., Hungary; 2 Department of Photosynthesis, Institute of Plant Physiology and Genetics, Bulgarian Academy of Sciences, 1113 Sofia, Acad. G. Bonchev Street, Bldg. 21, Bulgaria

## Abstract

The exact relationship between polyamine, abscisic acid and proline metabolisms is still poorly understood. In the present study, the effects of putrescine and abscisic acid treatments alone or in combination with polyethylene glycol-induced osmotic stress were investigated in young wheat plants. It was observed that abscisic acid plays a role in the coordinated regulation of the proline and polyamine biosynthetic pathways, which compounds are related to each other through a common precursor. Abscisic acid pre-treatment induced similar alteration of polyamine contents as the osmotic stress, namely increased the putrescine, but decreased the spermidine contents in the leaves. These changes were mainly related to the polyamine cycle, as both the synthesis and peroxisomal oxidation of polyamines have been induced at gene expression level. Although abscisic acid and osmotic stress influenced the proline metabolism differently, the highest proline accumulation was observed in the case of abscisic acid treatments. The proline metabolism was partly regulated independently and not in an antagonistic manner from polyamine synthesis. Results suggest that the connection, which exists between polyamine metabolism and abscisic acid signalling leads to the controlled regulation and maintenance of polyamine and proline levels under osmotic stress conditions in wheat seedlings.

## Introduction

There is increasing evidence that plant responses to unfavourable environment can be modulated by various plant hormones and plant growth regulators. Polyamines (PAs) are also involved in plant stress responses and tolerance. The most abundant PAs in plants are putrescine (PUT), spermidine (SPD), and spermine (SPM), which can be found in relatively high amount. PUT is synthesized by the decarboxylation of ornithine, catalysed by ornithine decarboxylase (ODC), or indirectly by the decarboxylation of arginine by arginine decarboxylase (ADC), via agmatine. After that, SPD and SPM are produced by the sequential addition of aminopropyl moieties to the putrescine skeleton through enzymatic reactions catalysed by SPD and SPM synthases (SPDS and SPMS). The donor of the aminopropyl groups is decarboxylated S-adenosyl-methionine, which is synthesized from S-adenosyl-methionine by S-adenosyl-methionine decarboxylase (SAMDC). PAs are catabolised by diamine oxidases (DAOs) and polyamine oxidases (PAOs)^1^.

Increased PA accumulation is accompanied by an increase in the activity of PA synthesis enzymes and expression of their genes have been reported in several plant species under stress conditions. In addition, investigations on the genetic modification of their synthesis enzymes together with exogenous applications of PAs suggest that the level of tolerance may correlate with the elevated cellular PA content^2^. The functions and the roles of the individual PAs in plant stress processes are diverse and sometimes contradictory. PAs are double face molecules, act as direct ROS scavenging and influence antioxidant activity at molecular and gene expression level. However, they are also ROS sources due to their apoplastic catabolism and the PA-cycle^1^ (Suppl. Fig. 1). Compared to other hormones, PAs are often present in high concentrations; according to this, changes in their metabolism may cause shift in the cellular metabolism, which presumes that the existence of fine regulation and tuning is necessary^3^.

The plant hormone abscisic acid (ABA) regulates several physiological processes and may also induce tolerance to various abiotic stresses^4^. The involvement of ABA in drought stress tolerance has been studied extensively at physiological and molecular levels, and a sharp increase in its endogenous level was also detected during water deficit conditions^5^. ABA plays a central role for improvement of plant drought resistance not only by its effect on stomatal closure but also by inducing activities or gene expression of antioxidant enzymes^6^. ABA also increases the gene expression level of the PA biosynthesis genes^7^. On the other hand, overexpression of the genes of PA synthesis enzymes, such as *ADC*, *SAMDC* or *SPMS*, resulted in increased ABA biosynthesis due to the higher expression level of 9-cis-epoxycarotenoid dioxygenase (*NCED*). In addition, ABA-related transcription factors were also upregulated^8^. These results suggest that there is a positive feedback loop between ABA and PAs.

Proline also accumulates in many plant species in response to environmental stress^9–11^ and acts as a major reservoir of energy and nitrogen, which can be utilized under stress conditions. Increased proline contents were detected not only upon stress conditions but also after ABA and PA treatments^12–18^. The facts that proline accumulation is mediated by both ABA-dependent and ABA-independent signalling pathways and that ABA modulates proline synthesis both on transcriptional level through induction of gene expression of Δ_1_-pyrroline-5-carboxylate synthase (*P5CS*) and on post-transcriptional level by stabilizing the *P5CS* transcript are well-studied^19^. However, correlation between proline accumulation and abiotic stress tolerance in plants is not always acknowledged^20^. Furthermore, the fact that increased PA levels – resulted from exposure to abiotic stress, exogenous PA treatments or genetic manipulation – led to increased proline content is interesting, especially if we take into consideration that their synthesis shares a common precursor, glutamate^3^ (Suppl. Fig. 1). Pronounced contribution of PUT degradation – by DAO – to proline accumulation has also been reported^21^. Although several abiotic stresses have been reported to stimulate PA oxidation, the precise role of PA catabolism in the plant response to environmental stress remains elusive.

According to the relationships described above, understanding the regulation of PA metabolism in plants is of major interest. However, the exact relationship between PA, ABA and proline metabolism is still poorly understood. The main aim of the present experiment was to find answers to the following questions: 1. How does ABA treatment influence the PA metabolism, and vice versa: how do PAs influence the ABA level in wheat? 2. Do specific steps in the PA metabolism respond differently under control or mild osmotic stress conditions? 3. What relationship exists between PAs and proline content and synthesis? The answers to these questions may increase the understanding of the function of PA metabolism in relation with ABA and proline, which compounds have well known role in drought or osmotic stress responses.

## Results

Before the present work demonstrated in this paper a pilot experiment was carried out using 2 wheat genotypes including TC33 and a winter wheat variety Mv Hombár from Agricultural Institute, Centre for Agricultural Research, Hungarian Academy of Sciences, Martonvásár. These preliminary results revealed that 0.15 mM ABA for 1 day provided protection against osmotic stress induced by 15% PEG manifested in the gas exchange parameters (data not shown). ABA treatment alone did not only influence the proline content, but the PA content was also changed (data not shown). As changes induced in the PA and proline contents were similar in the two genotypes, for further experiment TC33 was chosen for the present experiment, as this was relatively drought sensitive. In order to clarify influence of ABA or PEG on PA metabolism, as an additional treatment, PUT was also used. PA metabolism is linked with proline metabolism, so another question of the present work was to reveal how changes in PA content influence proline synthesis.

### Gas exchange parameters and relative water contents

In order to characterize the effects of ABA, PUT and PEG treatments on the physiological status of control and PEG-treated wheat (TC 33) plants, gas exchange parameters were determined in all of the treatments, while relative water content was measured at the end of the experiment after 5 days with or without PEG treatment. 1d ABA pre-treatment induced significant decrease in P_n_ due to a pronounced stomatal closure, which was in parallel with the decrease of the intracellular CO_2_ concentration indicating the continuation of photosynthesis, while 1d PUT pre-treatment did not influence the gas exchange parameters. After the 5 days of recovery period, these differences in the P_n_ and C_i_ parameters mainly disappeared in ABA-treated (ABA + 5d) plants compared to the same day of control (C + 5d) where slightly lower stomatal conductance and transpiration have been still detected (Table 1). PEG treatment alone decreased the g_s_ and E parameters as a mild osmotic stress, and similar values were detected in the combined treatments (ABA + 5dPEG and PUT + 5dPEG) (Table 1). ABA and PUT pre-treatments did not influence the RWC while PEG treatment either alone or in combination with PUT decreased it (Table 1).

**Table 1:** Effects of 1-day 0.15 mM abscisic acid (ABA) or 0.5 mM putrescine (PUT) pre-treatments followed by 5 days of recovery period or 15% polyethylene glycol (PEG) treatments on gas exchange parameters (Pn: CO_2_ assimilation rate; Ci: intracellular CO_2_ concentration; gs: stomatal conductance and E: transpiration) and relative water content (RWC) of the leaves of wheat plants.

Treatments	Pn (μmol CO_2_ m^−2^ s^−1^)	Ci (μmol CO_2_ mol^−1^)	gs (mol CO_2_ m^−2^ s^−1^)	E (mmol H_2_O m^−2^ s^−1^)	RWC
C	7.592 ± 0.83 b	298 ± 17 b	0.158 ± 0.033 ef	2.122 ± 0.355 d	
ABA	5.03 ± 1.683 a	16 ± 5 a	0.018 ± 0.014 a	0.267 ± 0.213 a	
Put	9.04 ± 1.067 b	274 ± 18 b	0.15 ± 0.011 e	1.867 ± 0.197 d	
C + 5d	6.616 ± 0.86 a	351 ± 19 c	0.238 ± 0.02 g	1.933 ± 0.153 d	93.55 ± 1.50 cd
ABA + 5d	5.8 ± 0.889 a	329 ± 13 c	0.144 ± 0.031 def	1.296 ± 0.255 c	93.68 ± 2.35 bcd
PUT + 5d	5.853 ± 0.15 a	359 ± 24 c	0.204 ± 0.022 fg	2.033 ± 0.462 d	94.76 ± 1.37 d
C + 5dPEG	5.037 ± 0.574 a	313 ± 19 bc	0.083 ± 0.019 bc	0.917 ± 0.196 b	89.3 ± 3.74 ab
ABA + 5dPEG	5.733 ± 0.75 a	317 ± 26 bc	0.114 ± 0.043 cdf	1.13 ± 0.294 bc	92.66 ± 1.56 bc
PUT + 5dPEG	5.31 ± 0.41 a	314 ± 1.4 bc	0.100 ± 0.021 cd	1.01 ± 0.155 bc	90.39 ± 1.32 a

Data represent mean values ± SD, n = 10. Different letters indicate significant differences between the treatments at P < 0.05.

### ABA content and synthesis

**Figure 1:**
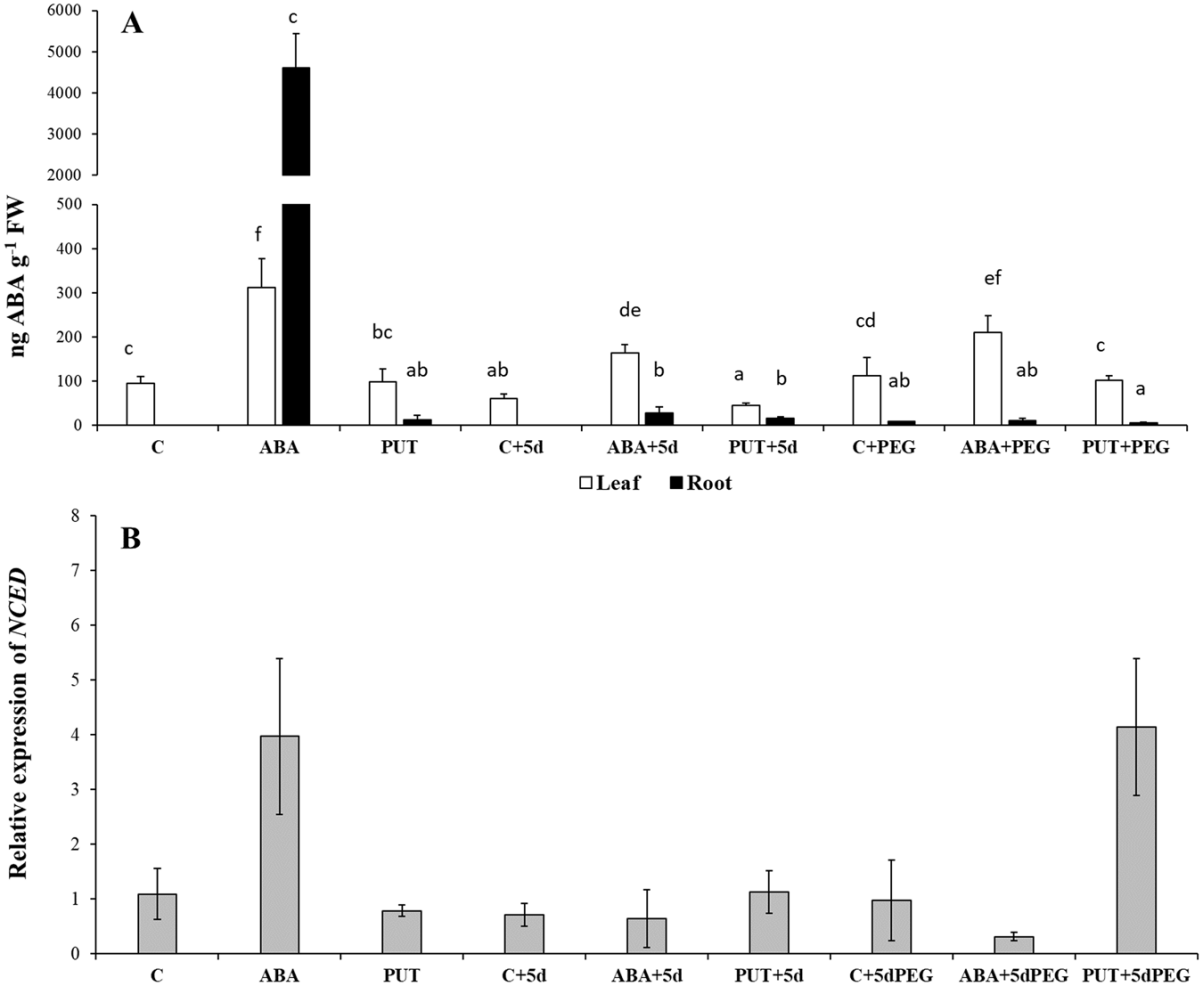
Effects of 1 day 0.15 mM abscisic acid (ABA) or 0.5 mM putrescine (PUT) pre-treatments followed by 5 days of recovery period or 15% polyethylene glycol (PEG) treatments on the abscisic acid content (**A**) in the leaves and roots, and gene expression level of 9-cis-epoxycarotenoid dioxygenase (NCED: **B**) in the leaves of wheat plants. Data represent mean values ± SD, n = 5. Different letters indicate significant differences between the treatments at P < 0.05.

Despite the thorough root washing high ABA content was detected in case of the root after 1-day ABA pre-treatment. However, increased ABA content was found not only in the roots but also in the leaves, and the increased ABA levels were still detected both in the leaves and roots of ABA + 5d wheat plants compared to the control plants (Fig. 1A). PUT pre-treatment did not influence the ABA content in the leaves, but in the roots where in the control ABA contents could not be detected, definite peaks were identified (Fig. 1A). PEG treatment alone also increased the ABA content especially in the leaves, and similar changes were induced in the PUT + 5dPEG-treated plants. Under osmotic stress conditions, the highest ABA accumulation in the leaves was found in the case of the ABA + 5dPEG treatment. The expression level of 9-cis-epoxycarotenoid dioxygenase (*NCED*), the gene encoding the key enzyme of ABA biosynthesis showed low expression level in the leaves and increased only in the case of ABA and PUT + PEG treatments (Fig. 1B).

### PA metabolism

**Figure 2:**
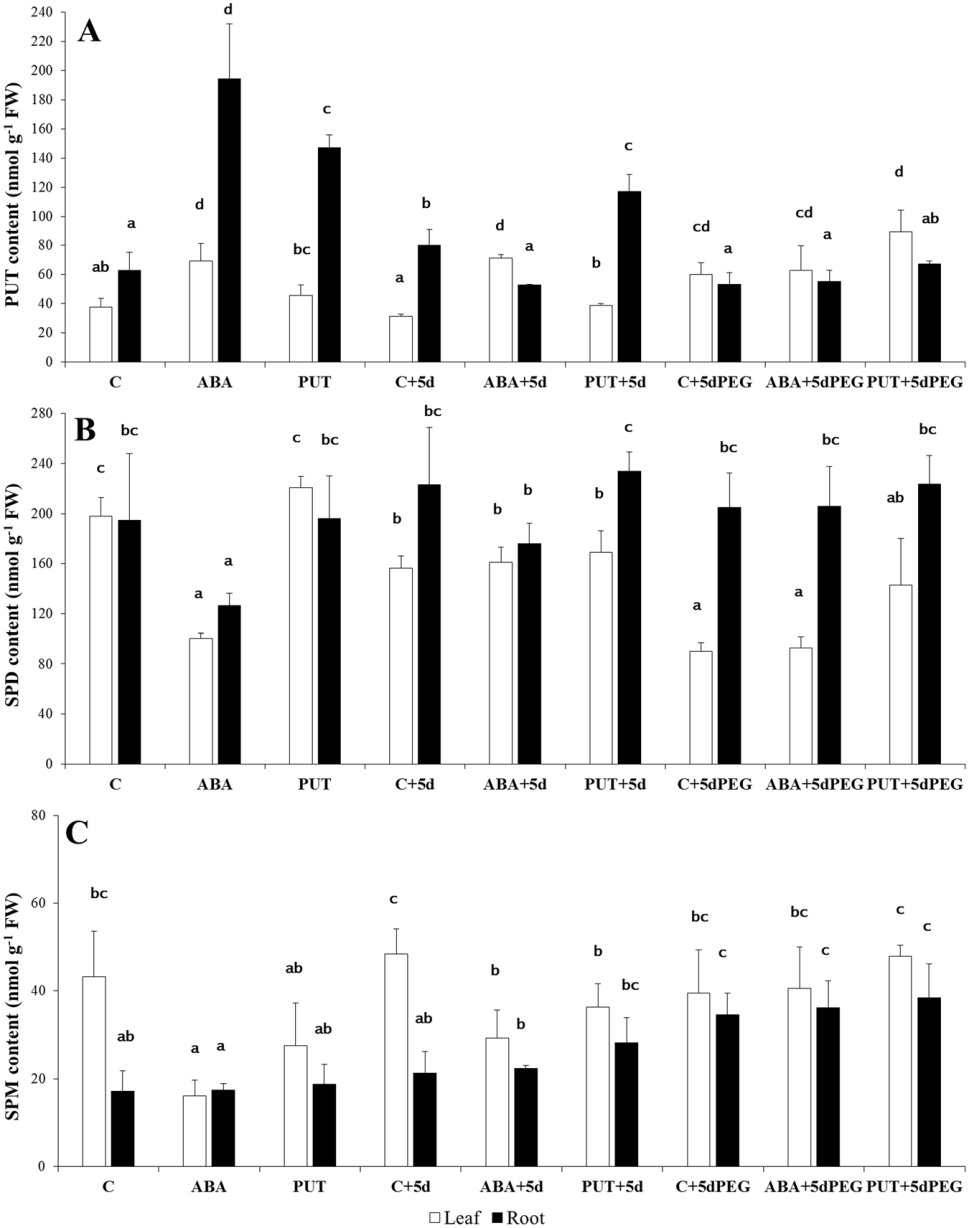
Effects of 1 day 0.15 mM abscisic acid (ABA) or 0.5 mM putrescine (PUT) pre-treatments followed by 5 days of recovery period or 15% polyethylene glycol (PEG) treatments on the free polyamine contents, namely putrescine (PUT: **A**), spermidine (SPD: **B**) and spermine (SPM: **C**) in the leaves (white bars) and roots (black bars) of wheat plants. Data represent mean values ± SD, n = 5. Different letters indicate significant differences between the treatments at P < 0.05.

Not surprisingly, 1d PUT treatment caused PUT accumulation in the roots, but this concentration with such duration was not enough for the induction of root to shoot translocation or significant increase in the content of higher PAs (SPD or SPM) (Fig. 2A–C). According to this, the PUT/(SPD + SPM) ratio increased in the roots (C: 0.30 and PUT:0.69) but did not change remarkably in the leaves (C: 0.16 and PUT: 0.18). Increased PUT content was still detected after the 5 days of recovery period without any treatment, especially in the roots (PUT + 5d), with 0.33 and 0.45 PUT/(SPD + SPM) ratio for C + 5d and PUT + 5d, respectively. In the leaves, similar values were found as in C and PUT; 0.15 for C + 5d and 0.19 for PUT + 5d. In contrast to this, ABA treatment (ABA) induced more extensive changes in PA contents. After 1d ABA treatment, PUT accumulation was in parallel with a significant decrease in the SPD content both in the leaves and roots, while SPM level decreased in the leaves. After the recovery period, only increased leaf PUT and decreased leaf SPM content was detected in ABA + 5d plants compared with the same day control (C + 5d) (Fig. 2A,C). These changes also influenced the PUT/(SPD + SPM) ratio, 0.59 for ABA and 0.38 for ABA + 5d in the leaves, and 1.35 for ABA and 0.27 for ABA + 5d in the roots.

15% PEG treatment for 5 days induced pronounced shift in the ratio of PUT/(SPD + SPM) as it increased (0.46) in a similar way in the leaves as ABA or ABA + 5d treatments, while it decreased (0.22) in the roots as it was found in the case of ABA + 5d. In the background of these alterations, increased leaf PUT and root SPM but decreased leaf SPD contents were detected (Fig. 2A–C). In the combined treatments (ABA + 5dPEG or PUT + 5dPEG), similar patterns of the PA content and ratio were found as it was described in the case of C + 5dPEG treatment.

**Figure 3:**
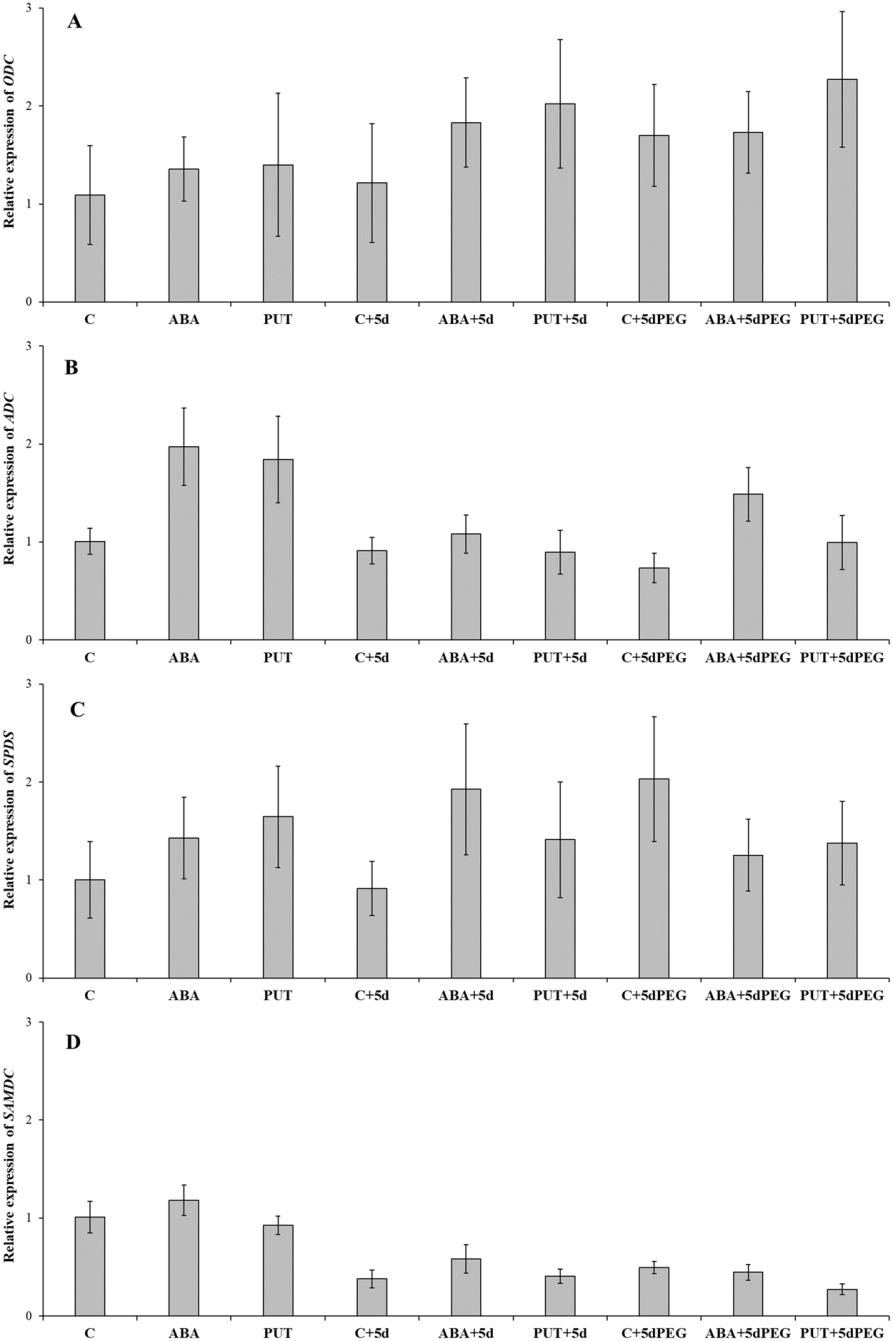
Changes in the gene expression of the arginine decarboxylase (ADC: **A**), ornithine decarboxylase (ODC: **B**), spermidine synthase (SPDS: **C**) and S-adenosyl-methione decarboxylase (SAMDC: **D**) genes in the leaves of wheat plants after 1-day of 0.15 mM abscisic acid (ABA) or 0.5 mM putrescine (PUT) pre-treatments followed by 5 days of recovery period or 15% polyethylene glycol (PEG) treatments. The relative gene expression values were determined with the *ΔΔ*Ct method. All reactions were performed in triplicate.

The applied treatments slightly influenced the PA synthesis genes in the leaves of young wheat plants. Both pathways of the PUT biosynthesis were active, as the gene expression level of *ODC* and *ADC* was detected in all cases (Fig. 3A,B). Only ABA, PUT and ABA + 5dPEG treatments could increase the level of *ADC* transcript. The expression level of *SPDS* and *SAMDC*, which are responsible for synthesis of higher PAs did not show remarkable changes (Fig. 3C,D).

**Figure 4:**
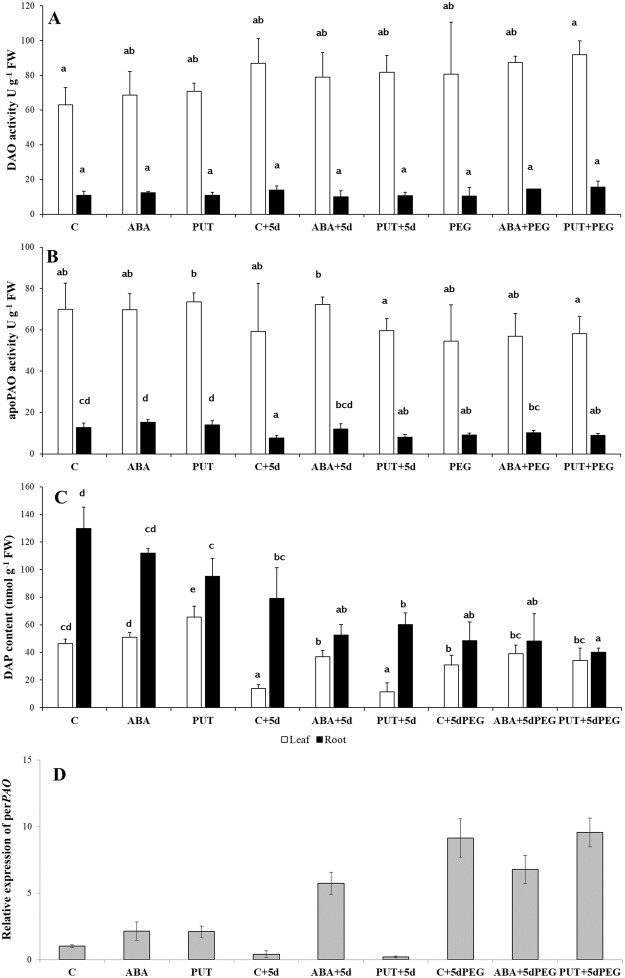
Effects of 1 day 0.15 mM abscisic acid (ABA) or 0.5 mM putrescine (PUT) pre-treatments followed by 5 days of recovery period or 15% polyethylene glycol (PEG) treatments on the activities of apoplastic diamine oxidase (DAO: **A**) and polyamine oxidase (apoPAO: **B**) and 1,3-diaminopropane (DAP: **C**) content in the leaves (white bars) and roots (black bars) of wheat plants. Data represent mean values ± SD, n = 5. Different letters indicate significant differences between the treatments at P < 0.05. Changes in the gene expression of the peroxisomal polyamine oxidase (perPAO: **D**) gene in the leaves of wheat plants. The relative gene expression values were determined with the *ΔΔ*Ct method. All reactions were performed in triplicate.

The main PA catabolic process is localised in the apoplast and exerted through DAO and PAO, the former showing a strong preference for diamines (PUT and cadaverin), while the latter only oxidizes higher PAs (SPD and SPM). Apoplastic PAOs (apoPAO), which are responsible for this terminal catabolism of PAs, oxidize SPD and SPM to 1,3- diaminopropane (DAP). Although none of the applied treatments influenced the DAO or apoPAO activities in wheat plants significantly (Fig. 4A,B). Increased DAP level was detected in the leaves of PUT-treated plants compared to the same-day control (C), but similar amount was found after a recovery period in PUT + 5d compared to C + 5d. ABA + 5d, C + 5dPEG, ABA + 5dPEG and PUT + 5dPEG also increased it in the leaves in comparison with the control of the same day (Fig. 4C). In parallel with these, the gene encoding the peroxisomally localised PAO enzyme responsible for the back-conversion of SPM to SPD, and SPD to PUT also increased in the cases of ABA + 5d and all the PEG treatments (C + 5dPEG, ABA + 5dPEG, PUT + 5dPEG) (Fig. 4D).

### Proline metabolism

**Figure 5:**
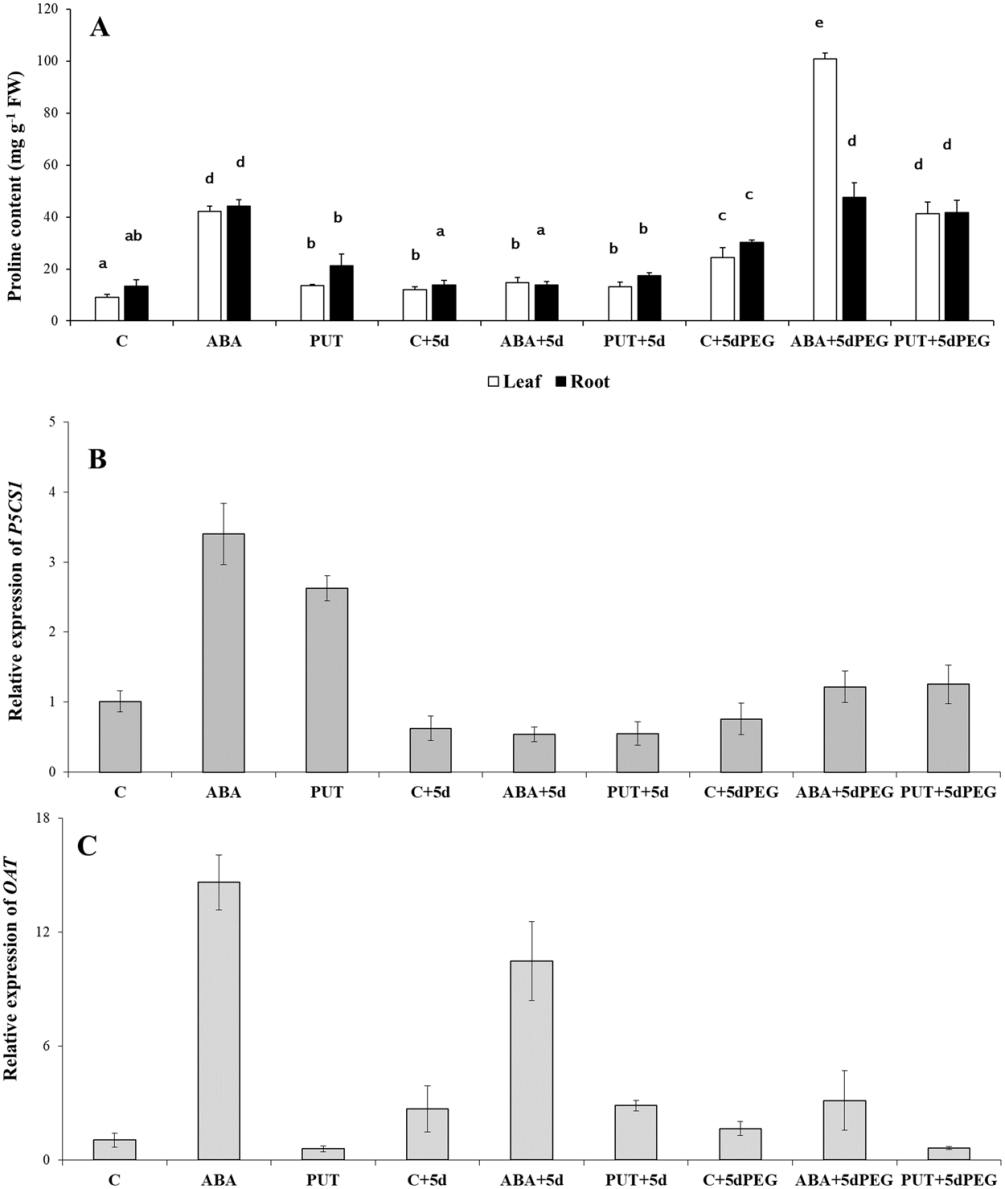
Effects of 1 day 0.15 mM abscisic acid (ABA) or 0.5 mM putrescine (PUT) pre-treatments followed by 5 days of recovery period or 15% polyethylene glycol (PEG) treatments on the proline contents (**A**) in the leaves (white bars) and roots (black bars) of wheat plants. Data represent mean values ± SD, n = 5. Different letters indicate significant differences between the treatments at P < 0.05. Changes in the gene expression of the Δ1-pyrroline-5-carboxylate synthase (P5CS1: **B**) and ornithine aminotransferase (OAT: **C**) genes in the leaves of wheat plants. Changes in the gene expression of the peroxisomal polyamine oxidase (perPAO: **D**) gene in the leaves of wheat plants. The relative gene expression values were determined with the *ΔΔ*Ct method. All reactions were performed in triplicate.

1d ABA treatment induced high accumulation of proline both in the leaves and roots of wheat plants, while PUT caused only slight increase in the leaf proline content (Fig. 5A). However, these differences disappeared after the 5 days of recovery period. Although PEG-induced stress alone increased the level of proline in the leaves and roots significantly, in the combined treatments, as an additive effect of the plant growth regulator pre-treatments followed by PEG treatment, higher proline accumulations were found. The highest increment was detected in the leaves of ABA + PEG-treated plants (Fig. 5A).

The genes involved in proline synthesis responded differently to the applied treatments. The expression level of *P5CS1* gene, which encodes Δ^1^-pyrroline-5-carboxylate synthetase that catalyses the synthesis of proline from glutamate, showed that especially ABA and PUT pre-treatments induced it, but these changes disappeared after the recovery period, while ABA + 5dPEG and PUT + 5dPEG, where high proline accumulation was found, caused only a slight increment compared to the same day of control (Fig. 5B). In comparison, *OAT* gene, which encodes ornithine aminotransferase (the enzyme catalysing the synthesis of proline from ornithine), was induced after ABA pre-treatment, and this induction could also be observed after the recovery period. Interestingly, PUT + 5dPEG decreased it compared to the same day of control (Fig. 5C).

## Discussion

In the present work, the effect of ABA, PEG treatments and their combinations on PA metabolism were investigated in order to reveal the interactions between ABA and PAs in stress responses with special regard to their relationship with proline metabolism. In order to exclude and distinguish the effect of increased PUT level, PUT treatment alone was also applied. In addition, the effects of 1 d ABA or PUT treatments were also investigated after a 5-day-recovery period for further differentiation between the effects of ABA and PUT compared to the PEG treatment induced osmotic stress.

### ABA-PA relation

Recent results have shown that exogenous PUT increases the ABA content and the *NCED* transcript level in the leaves of tomato under chilling stress^22^. In the present case, although PUT treatment did not influence the ABA content and the gene expression level of *NCED* in the leaves, it caused slight but clear increase in root ABA content of young wheat plants, which was in parallel with pronounced root PUT accumulation. It has been demonstrated that rapid increase in PUT levels is required for ABA accumulation in response to low temperature in *Arabidopsis*, and the gene expression level of *NCED* was also increased by PUT addition^23^. In the present experiment the gene expression of *NCED* increased only in the leaves of ABA- and PUT + PEG-treated plants. However, other studies showed that the expression level of *NCED* may depend on the genotype; and the level of the expression is not always in correlation with changes in the ABA content under PEG-induced osmotic stress conditions^24^.

In contrast to the effect of PUT on ABA content, ABA treatment had powerful effect on PA metabolism. The most characteristic changes were the increase in PUT and the decrease in SPD contents of ABA-treated plants. In addition, after the recovery period (ABA + 5d), the PUT-increasing and SPM-decreasing effects of ABA were still detected. Correlation analyses also revealed that a positive relationship existed between ABA and PUT, and ABA and DAP, but negative relationship was found between ABA and SPD or SPM contents (Table 2). Results also indicated that the degree of PUT accumulation was lower than the depletion in SPD content of ABA-treated plants. However, these changes did not result from decreased gene expression level of *SPDS* or *SAMDC* in the leaves, indicating that not the modification on the synthesis side is responsible for the observed decrease in the level of SPD and SPM. The accumulation of PUT in the wheat plants was in parallel with increased *ADC* gene expression in the case of ABA and PUT treatments. These results suggest that the exogenously applied PUT was not only taken up by the wheat plants but also induced its *de novo* synthesis in the leaves. Our results are in accordance with earlier findings when ABA also induced increase in PUT content of 3-day-old wheat seedling, which could be inhibited by α-difluoromethylarginine (an inhibitor of ADC) or α-difluoromethylornithine (an inhibitor of ODC) in the shoots^25^. Exogenous ABA also increased the PUT contents in chickpea^26^, while ABA has been reported to trigger PA synthesis through a transcriptional activation of genes encoding SPDS in maize^27^. Under the present experimental conditions a positive correlation has also been found between ABA content and gene expression level of *ADC* and *SAMDC* (Table 2).

**Table 2:** Correlation analysis of the investigated parameters in the leaves of wheat plants after 1-day 0.15 mM abscisic acid or 0.5 mM putrescine pre-treatments followed by 5 days of recovery period or 15% polyethylene glycol (PEG) treatment.

	PUT	SPD	SPM	DAP	DAO	apoPAO	*ADC*	*ODC*	*SPDS*	*SAMDC*	per***PAO***	ABA	*NCED*	PRO	*P5CS*	*OAT*
PUT	1															
SPD	−0,445*	1														
SPM	−0,104	0,112	1													
DAP	0,216	0,221	−0,411*	1												
DAO	0,047	−0,118	0,114	−0,325	1											
apoPAO	0,0005	0,409*	−0,14	0,245	−0,434*	1										
*ADC*	0,073	0,124	−0,499*	0,746**	−0,436*	0,287	1									
*ODC*	0,203	−0,183	−0,08	−0,261	0,619**	−0,269	−0,384	1								
*SPDS*	0,489*	−0,115	−0,379	0,225	0,028	0,073	0,046	0,312	1							
*SAMDC*	−0,065	0,154	−0,667**	0,666**	−0,44*	0,334	0,641**	−0,355	0,017	1						
per*PAO*	0,649**	−0,526**	0,19	0,115	0,147	−0,366	−0,249	0,403	0,536*	−0,312	1					
ABA	0,478*	−0,526**	−0,493**	0,412*	−0,331	0,196	0,578**	−0,079	0,193	0,555**	0,221	1				
*NCED*	0,447*	−0,236	−0,216	0,128	0,1	0,135	0,126	0,153	−0,018	0,185	0,118	0,315	1			
PRO	0,467*	−0,653**	−0,058	0,175	0,238	−0,233	0,199	0,248	−0,015	−0,087	0,487*	0,513*	0,134	1		
*P5CS*	0,071	0,061	−0,572**	0,69**	−0,138	0,333	0,749**	−0,085	0,039	0,783**	−0,112	0,523**	0,535**	0,181	1	
*OAT*	0,259	−0,291	−0,731**	0,128	−0,238	0,425	0,389	−0,078	0,269	0,423	−0,236	0,727**	0,102	0,078	0,222	1

Significant correlations at 0.05 level were highlighted in bold. Investigated parameters: contents of ABA: abscisic acid; DAP: 1,3-diaminopropane; PUT: putrescine; SPD: spermidine and SPM: spermine; enzyme activities of apoPAO: apoplastic polyamine oxidase and DAO: diamine oxidase; gene expression levels of ADC: arginine decarboxylase; NCED: 9-cis-epoxycarotenoid dioxygenase; OAT: ornithine aminotransferase; ODC: ornithine decarboxylase; perPAO: peroxisomal polyamine oxidase; P5CS: Δ1-pyrroline-5-carboxylate synthase; SAMDC: S-adenosyl-methione decarboxylase; SPDS: spermidine synthase. *Correlation is significant at the 0.05 level (2-tailed). **Correlation is significant at the 0.01 level (2-tailed).

ABA treatment enhanced the activity of apoPAO and also the expression level of gene encoding apoPAO in maize^28^. In *Arabidopsis*, ABA-induced perPAO enhances the back-conversion pathway of PAs^29^. Not only increased activities of PAOs, but also induced PA exodus (transport of PAs from the cytosol to the apoplast) were detected in *Vitis vinifera* after ABA treatment^30^. In contrast to these, in the present study, ABA or PUT treatments did not influence the activities of DAO and apoPAO remarkably, which are localized in the apoplast and have a role in the terminal catabolism of PUT and SPD/SPM, respectively. However, the gene expression of the per*PAO* was induced after ABA treatments indicating that the interconversion of SPD/SPM to PUT occurred in the leaves. These results suggest that ABA modulates PA metabolism in a complex way at transcriptional level and the result of the induced changes in back-conversion displayed in the observed PA pattern.

### PA-osmotic stress

ABA plays a key role in the responses of plants to drought or osmotic stress conditions, and its biosynthesis may be induced firstly in the roots and then the hormone can be readily transported in the xylem into the leaves. Changes in the PA metabolism under osmotic stress conditions have also been extensively studied for a long time^31–33^. However, results are sometimes controversial and especially its relationship with the ABA signalling is less understood. Interestingly, ABA-induced PA pattern in the wheat leaves was similar to that of PEG-treated plants, as 15% PEG for 5 days increased PUT and decreased SPD content. However, significant changes could not be detected in the roots. In addition, PEG either alone or in the cases of combined treatments (ABA + 5dPEG and PUT + 5dPEG) did not induce remarkable changes in the gene expression pattern of PA synthesis enzymes, except for the *ADC*, as its transcript level was slightly increased by the ABA + 5dPEG treatment. Although osmotic stress alone or in the combined treatments did not affect the apoplastic DAO or PAO activities, but increased leaf DAP content and the gene expression level of the peroxisomal *PAO*.

Partly similarly to the present experiment, 0.1 mM ABA or 15% PEG applied for 3 weeks did not influence PUT content, but it induced a continuous decrease in the SPD level in parallel with increased SPM content in the leaves of wheat plants^34^. However, earlier studies focusing on changes in the activities or gene expression levels of the enzymes, which are involved in the metabolism of PAs are limited. The analysis of PA content in PA synthesis mutant *Arabidopsis* during drought stress revealed that drought induced strong metabolic canalization of SPM to PUT due to the PA back-conversion pathway, but not to the terminal catabolism of SPM^35^. Similarly to water deficit, ABA treatment increases stomatal resistance and decreases transpiration rate as it was also found in the present experiment. PUT and SPD at 0.5 mM for 7 days were also reported to induce stomatal closure in wheat^18^. Moreover, it was shown that DAO in *Vicia faba*, while PAO in *Vitis vinifera* are involved in the ABA-induced stomata closure due to the subsequent H_2_O_2_ production^30,36^. In the present experiment, apoplastic DAO and PAO activities did not change despite the observed stoma closure. The findings that higher SPD degradation can occur without increase in PAO activity is consistent, since it has also been suggested that instead of the induction of the already high apoplastic DAO/PAO activity, rather the controlled PA exodus is responsible for the regulation of cellular PA levels^37^. In the present experiment, increased per*PAO* gene expression was found in the leaves of PEG treated plants.

The gene expression level of the PA synthesis enzymes were not inhibited, while DAP and PUT contents increased; in addition, the transcript level of per*PAO* also increased under osmotic stress conditions, suggesting that both the terminal catabolism and the back-conversion may be involved in the reduction of SPD level. Correlation analyses also revealed that positive relationship exist between SPD content and enzyme activity of apoplastic PAO, but negative relationship between SPD content and the gene expression level of per*PAO* (Table 2). According to these, osmotic stress-induced changes in PA content may be the resultant of the complex alteration in synthesis, exodus, degradation and back-conversion.

### PA-proline

Increase in proline content is considered as a drought-injury sensor^38^. Nevertheless, it is a long-standing question how proline accumulation and metabolism may be modified during the development of drought tolerance^39^. Thus it is important to understand the connections between PA and proline metabolisms.

It has been shown that up-regulation of PUT biosynthesis leads to widespread metabolic redistribution^40,41^. As biosynthesis of PAs and proline use glutamate as a common precursor, considerable changes in the pool of PAs could cause a shift between the synthesis pathway of PA and proline. Diversion of ornithine into PA biosynthesis does not only influence its biosynthesis from glutamate but also affects the arginine and proline biosynthesis. However, it is not always clear which pathway is involved in increased proline biosynthesis directly from glutamate by P5CS or from ornithine by OAT^40^. In high PUT-producing transgenic *Arabidopsis*, it was revealed that production of proline is regulated independently from the glutamate-ornithine-arginine pathway, which latter pathway is regulated rather at enzymatic than at transcriptional level. However, the regulation of the flux of glutamate into PAs or proline under stress conditions, when increased synthesis of PUT occurred, is still enigmatic. Furthermore, not only the syntheses of PA and proline are linked but PA catabolism has also been shown to be closely related to proline accumulation, which was associated with a rapid decrease in PUT and SPD levels and some increase in DAP, and the CuAO inhibitor inhibited the accumulation of proline^32^. Correlation analyses revealed that under the present experimental conditions the *OAT* transcript level was in positive relationship with ABA contents, and negative relationship with SPM, while the *P5CS* transcript level was also in a negative relationship with the SPM content, but in a positive relationship with DAP and ABA contents and gene expression levels of *ADC, NCED* and *SAMDC* (Table 2).

In the present experiment, both ABA and PUT treatments, as well as osmotic stress conditions increased the level of proline, with the highest accumulation in the leaves of ABA + 5dPEG-treated plants. However, proline accumulation was in positive correlation with PUT content, but in negative correlation with SPD content (Table 2), direct cause and effect relationship was not responsible for the observed increase of proline. Despite the similar effect of ABA and osmotic stress on PA pattern, different effect of them on proline synthesis was observed in wheat leaves. ABA or PUT pre-treatments resulted in PUT accumulation and induced the activation of *P5CS* gene expression, but only ABA pre-treatment increased the expression level of *OAT* gene. Although proline accumulation was observed after 5 days of PEG treatment, osmotic stress did not influence either of the proline biosynthesis pathways on the same day. However, the fact that greatest proline content was measured in the plants treated with ABA + 5dPEG could not be explained by only the actually detected changes in the gene expression of *OAT* or *P5CS1*. These results suggest that ABA-induced increased gene expressions of *OAT* and *P5CS1* were responsible for proline accumulation, which changes were not related directly to the excess of endogenous PUT, as PUT pre-treatment induced only *P5CS1* and resulted in lower increase in proline content also in the case of PUT + 5dPEG treatment. In addition, it has been reported that P5CS is subjected to feedback inhibition by increased proline content^38^. According to these, production of proline was partly regulated independently and not in an antagonistic manner from the PA synthesis. Nevertheless, as proline accumulation was in positive correlation with increased per*PAO* gene expression level (Table 2), it cannot be excluded that the PA catabolism and the PA cycle is related to proline synthesis.

## Conclusions

Our results suggest that a connection exists between PA metabolism and ABA signalling leading to a controlled regulation and maintenance of the SPD and SPM levels under osmotic stress in wheat seedlings. ABA modulates PA metabolism in a complex way at transcriptional level and the results of catabolism and/or back-conversion displayed in the observed PA pattern both under control condition and during osmotic stress. Despite the similar effects of ABA and osmotic stress on PA pattern, different effects on proline synthesis were observed in the leaves. Synthesis of proline and PAs were partly regulated independently and not antagonistically; in addition, the PA catabolism and the PA cycle is suggested to be related to proline synthesis (Suppl. Fig. 1). As a further prospect, investigation on the function and regulation of different members of the PA oxidase families in wheat may help for the better understanding of the relation of proline and PA metabolisms.

## Materials and methods

### Plant material and growth conditions

In the present experiment, a spring wheat (*Triticum aestivum* L. TC33) (Thatcher-based near-isogenic line, TC33: Thatcher*6/P.I.58548) genotype was used. After 3 days of germination between moistened filter papers at 22 °C in the dark, seedlings were grown in modified Hoagland solution^42^ at 22/20 °C, 16/8-h light/dark periodicity and 75% relative humidity in a Conviron GB-48 plant growth chamber (Controlled Environments Ltd, Winnipeg, Canada). Plastic containers were planted with 12 wheat seedlings and placed in the growth chamber in a fully randomized manner. The photosynthetic photon flux density (PPFD) was 250 μmol m^−2^ s^−1^. The plant growth solution was changed every two days.

After 14 days of growth in modified Hoagland solution, the wheat plants were treated hydroponically with 0.15 mM ABA or 0.5 mM PUT for 24 h and part of the 15-day-old plants were sampled with the control from the same day (C: without any treatment). Concentrations were chosen based on our previous results, where 0.1 mM ABA pre-treatment for 2 days effectively decreased chilling injury in maize^43^ and where 0.5 mM PUT pre-treatment for 7 days alleviated PEG-induced osmotic stress in maize and wheat^18^, and these treatments also efficiently induced changes in the antioxidant enzyme activities and SA content. After a gentle but thorough root washing in distilled water twice, another part of the plants was divided into six groups. C, ABA and PUT pre-treated plants were either moved to control growth condition as a recovery period (C + 5d, ABA + 5d or PUT + 5d) or treated with 15% PEG 6000 as an osmotic stress for 5 days (C + 5dPEG, ABA + 5dPEG or PUT + 5dPEG). After 5 days of PEG treatment, the roots and youngest fully developed leaves were sampled for further analysis. The growth solution of the plants during the growing condition and PEG treatment was changed every 2^nd^ day. The duration of the PEG treatment was established based on previous results^9,44^.

### Gas exchange measurements

Gas exchange analyses were performed after 24 h pre-treatments or 5 days after PEG-induced osmotic stress treatment on the intact, last fully expanded leaves using a Li-6400 instrument (Li-Cor, Lincoln, USA). The reference level of CO_2_ was 380 μL L^−1^, and the light intensity was 250 μmol m^−2^ s^−1^. The gas exchange analysis was performed at room temperature; the air humidity was 50 ± 3% in both cases. The parameters of CO2 assimilation rate (P_n_), stomatal conductance (g_s_), intracellular CO_2_ concentration (C_i_) and transpiration (E) were determined at the steady-state level of photosynthesis.

### PA and 1,3-diaminopropane (DAP) analysis

The analysis was carried out as described earlier^44^, by 200 mg of leaves homogenized with 1 ml 0.2 M ice-cold perchloric acid and having left them to stand for 20 min on ice. The extract was centrifuged at 10000 g for 20 min and the supernatant was used. The most abundant PAs, namely PUT, SPD and SPM together with DAP – the product of SPD and SPM terminal catabolism – were analysed as dansylated derivatives via HPLC using a W2690 separation module and a W474 scanning fluorescence detector with excitation at 340 nm and emission at 515 nm (Waters, Milford, MA, USA).

### Diamine oxidase and PA oxidase enzyme activities

The activity of the diamine oxidase (DAO, EC 1.4.3.6.) and PA oxidase (apoPAO, EC 1.5.3.3.) enzymes was estimated by the method of Takács *et al*.^45^. Enzyme activity was expressed in nmol *Δ*^1^-pyrroline min^−1^ g^−1^ FW using an extinction coefficient of 1.86 × 10^3^ mol^−1^ cm^−1^.

### Gene expression analysis

Total RNA was extracted from fully developed leaf and root samples using TRI Reagent®. The samples were treated with DNase I and cleaned with a Direct-zol™ RNA MiniPrep Kit (Zymo Research, Irvine, CA, USA) according to the manufacturer’s instructions. cDNA synthesis was carried out by using M-MLV Reverse Transcriptase (Promega Corporation, Madison, WI, USA). Gene-specific primers and housekeeping primers (Suppl. Table 1)^46,47^, PCRBIO SyGreen Mix (PCR Biosystems, London, UK) and CFX96 Touch™ Real-Time PCR Detection System (Bio-Rad, Hercules, CA, USA) were used for quantitative real-time PCR reaction. The relative gene expression values were determined with the *ΔΔ*Ct method^48^. Ct values were normalized by the Ct values of housekeeping gene Ta30797 encoding phosphogluconate dehydrogenase. All reactions were performed in triplicate.

### Proline content

The proline content was determined on the basis of its reaction with ninhydrin, according to the Bates method^49^.

### Statistical analysis

Three independent repetitions were performed for each experiment, and representative data are presented. The results were the means of at least 5 replicates for measurements of the gas exchange parameters, spectrophotometric and HPLC determinations. The data were statistically evaluated using the standard deviation and *t-test* methods. The SPSS 17.0 statistical program (Statistical Package for the Social Sciences) was used to examine correlations between the parameters.

## Electronic supplementary material

Supplementary Information

## Data Availability

All data generated or analysed during this study are included in this published article (and its Supplementary Information files).
