# Supplementary material for: Interaction of polyamines, abscisic acid and proline under osmotic stress in the leaves of wheat plants
Source: Sci Rep. 2018 Aug 27;8:12839. doi: 10.1038/s41598-018-31297-6 (PMC6110863; doi:10.1038/s41598-018-31297-6)
Supplement: Supplementary file 1 — Supplementary Information [file 41598_2018_31297_MOESM1_ESM.pdf]

Interaction of polyamines, abscisic acid and proline under osmotic stress in the leaves of wheat plants

Magda Pál[1*], Judit Tajti[1], Gabriella Szalai[1], Violeta Peeva[2], Balázs Végh[1] and Tibor Janda[1]

[1]Department of Plant Physiology, Agricultural Institute, Centre for Agricultural Research, Hungarian Academy of Sciences, Hungary 2462 Martonvásár, POB 19.

[2] Department of Photosynthesis, Institute of Plant Physiology and Genetics, Bulgarian Academy of Sciences, Bulgaria 1113 Sofia, Acad. G. Bonchev Street, Bldg. 21

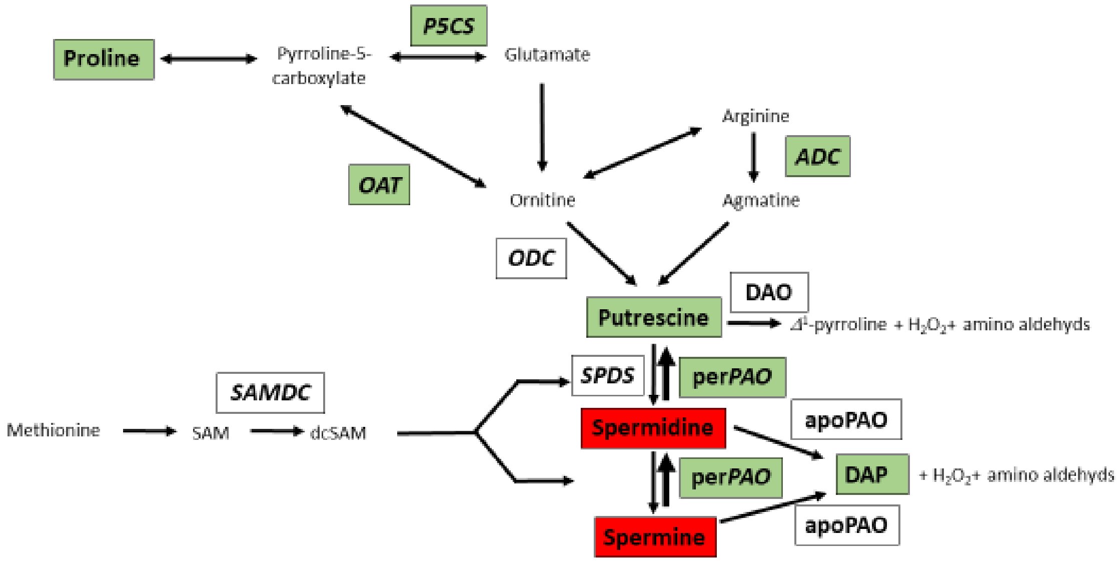

Suppl. Figure 1. Polyamine metabolism and its relationship with proline synthesis. Green boxes indicate ABA-induced increases, white boxes indicate no changes, while red boxes indicate the inhibitory effect of the 1 day 0.15 mM ABA treatment on polyamine metabolism and proline synthesis in wheat plants. *ADC*: arginine decarboxylase; apoPAO: apoplastic polyamine oxidase; DAO: diamine oxidase; DAP: 1,3-diaminopropane; *OAT*: ornithine aminotransferase; *ODC*: ornithine decarboxylase; PA: polyamine; per*PAO*: peroxisomal polyamine oxidase; PUT: putrescine; *P5CS*: Δ1-pyrroline-5-carboxylate synthase; *SAMDC*: S-adenosyl-methione decarboxylase; SPD: spermidine; *SPDS*: spermidine synthase; SPM: spermine.

Suppl. Table 1. Reference gene and target genes investigated in wheat plants using qRT-PCR.

Gene name	Primer sequences (5' → 3')		Reference
Ta30797 *(Similar to phosphogluconate dehydrogenase)*	Forward	GCCGTGTCCATGCCAGTG	Paolacci *et al.*, 2009
	Reverse	TTAGCCTGAACCACCTGTGC	
TaADC	Forward	TCTACCCCGTCAAGTGCAAC	own designed
	Reverse	GACGAGGCAGCTCATGGT	
TaODC	Forward	CGTGCGTGGAGGTGATAGG	own designed
	Reverse	AGCTGAGGGTGCCGTAGA	
TaOAT	Forward	TGATGATCGCTCGGCTTTACA	own designed
	Reverse	CAGTAGCACCCATTGTTGCAG	
TaP5CS1	Forward	AGGCTGGGTATGAGAGTGC	own designed
	Reverse	TAAGGCATCAGGTCGGGAC	
TaPAO	Forward	CCAGCCTCCAGCTCCGCAAC	Xiong *et al.*, 2017
	Reverse	GCCCAGCTCCTCCACCTCGTC	
TaSAMDC	Forward	ACAGCCTTCTCCACACAAGA	own designed
	Reverse	TCCAGACCAGTCATGCACA	
TaSPDS	Forward	AGGTATTCAAGGGTGGCGTG	own designed
	Reverse	TGGGTTCACAGGAGTCAGGA	
TaNCED	Forward	CCTCGAAGCCCAGCACTAAT	Gallé *et al.*, 2013
	Reverse	GAGAGCGAGAGGTCCAATGG	

References:

Gallé, Á. et al. Isohydric and anisohydric strategies of wheat genotypes under osmotic stress: biosynthesis and function of ABA in stress responses. J. Plant Physiol. 170, 1389-1399. (2013).

Paolacci, A.R., Tanzarella, O.A., Porceddu, E. & Ciaffi, M., Identification and validation of reference genes for quantitative RT-PCR normalization in wheat. BMC Molecular Biology 10, 11 (2009).

Xiong, H. et al. RNAseq analysis reveals pathways and candidate genes associated with salinity tolerance in a spaceflight-induced wheat mutant. Sci. Reports 7, 2731 (2017).
